# Assessment of intrafraction motion and its dosimetric impact on prostate radiotherapy using an in-house developed position monitoring system

**DOI:** 10.3389/fonc.2023.1082391

**Published:** 2023-07-14

**Authors:** Sankar Arumugam, Tony Young, Viet Do, Phillip Chlap, Christine Tawfik, Mark Udovitch, Karen Wong, Mark Sidhom

**Affiliations:** ^1^ Department of Medical Physics, Liverpool and Macarthur Cancer Therapy Centres and Ingham Institute, Sydney, NSW, Australia; ^2^ South Western Clinical School, University of New South Wales, Sydney, NSW, Australia; ^3^ Institute of Medical Physics, School of Physics, University of Sydney, Sydney, NSW, Australia; ^4^ Department of Radiation Oncology, Liverpool and Macarthur Cancer Therapy Centres, Sydney, NSW, Australia; ^5^ Department of Radiation Therapy, Liverpool and Macarthur Cancer Therapy Centres, Sydney, NSW, Australia

**Keywords:** prostate radiotherapy, online position monitoring, delivered dose assessment, treatment accuracy, standard linear accelerator, intrafraction motion

## Abstract

**Purpose:**

To implement an in-house developed position monitoring software, SeedTracker, for conventional fractionation prostate radiotherapy, and study the effect on dosimetric impact and intrafraction motion.

**Methods:**

Thirty definitive prostate radiotherapy patients with implanted fiducial markers were included in the study. All patients were treated with VMAT technique and plans were generated using the Pinnacle planning system using the 6MV beam model for Elekta linear accelerator. The target dose of 60 Gy in 20 fractions was prescribed for 29 of 30 patients, and one patient was treated with the target dose of 78 Gy in 39 fractions. The SeedTracker position monitoring system, which uses the x-ray images acquired during treatment delivery in the Elekta linear accelerator and associated XVI system, was used for online prostate position monitoring. The position tolerance for online verification was progressively reduced from 5 mm, 4 mm, and to 3 mm in 10 patient cohorts to effectively manage the treatment interruptions resulting from intrafraction motion in routine clinical practice. The delivered dose to target volumes and organs at risk in each of the treatment fractions was assessed by incorporating the observed target positions into the original treatment plan.

**Results:**

In 27 of 30 patients, at least one gating event was observed, with a total of 177 occurrences of position deviation detected in 146 of 619 treatment fractions. In 5 mm, 4 mm, and 3 mm position tolerance cohorts, the position deviations were observed in 13%, 24%, and 33% of treatment fractions, respectively. Overall, the mean (range) deviation of -0.4 (-7.2 to 5.3) mm, -0.9 (-6.1 to 15.6) mm, and -1.7 (-7.0 to 6.1) mm was observed in Left-Right, Anterior-Posterior, and Superior-Inferior directions, respectively. The prostate CTV D99 would have been reduced by a maximum value of 1.3 Gy compared to the planned dose if position deviations were uncorrected, but with corrections, it was 0.3 Gy. Similarly, PTV D98 would have been reduced by a maximum value of 7.6 Gy uncorrected, with this difference reduced to 2.2 Gy with correction. The V60 to the rectum increased by a maximum of 1.0% uncorrected, which was reduced to 0.5%.

**Conclusion:**

Online target position monitoring for conventional fractionation prostate radiotherapy was successfully implemented on a standard Linear accelerator using an in-house developed position monitoring software, with an improvement in resultant dose to prostate target volume.

## Introduction

Prostate cancer is one of the most common cancers globally, with GLOBOCAN 2020 reporting prostate cancer as having the third highest incidence worldwide out of the 36 cancer sites considered (1). For localized disease, external beam radiotherapy may be used as a treatment option. The use of modern and more conformal radiotherapy methods such as intensity modulated radiation therapy (IMRT) and volumetric modulated arc therapy (VMAT), along with improved imaging and tracking methods have enabled the reduction of side effects, enabling dose escalation and improving the therapeutic ratio (2–5).

Prostate localization cannot be reliant on skin marks and bony anatomy due to inter and intra fraction motion (6). The influence of interfraction motion may be reduced with daily image guidance and patient immobilization, as this motion is due to day to day prostate position changes in addition to variations in patient setup (7, 8). Intrafraction motion however, is due to internal organ motion during the actual treatment delivery, generally due to bowel and bladder filling during treatment (9–13). The effect of intrafraction motion is seen to be averaged out with increased fractionation as seen in conventional fractionation treatment regimens for the prostate, reducing the dosimetric effect (14). Additionally, treatment margins are applied to the target volume to ensure target dose coverage. However, this will result in surrounding organs at risk receiving an increased dose (10).

There are numerous real time monitoring systems available for use in radiotherapy. Early studies investigated the potential of using the implanted fiducial markers in the prostate with the portal imaging device on the Linear accelerator (linac) in addition to port films for tracking prostate motion (6, 7, 10, 15). Electromagnetic transponders (beacons) implanted in the prostate is another method which has been used for real time monitoring of the prostate, with commercial systems available for tracking of the implanted transponders during treatment (16–18). Tracking of the implanted fiducial markers using the kilovoltage imaging available on linear accelerators has also been used to monitor the prostate motion during treatment (19–23). Cine mode Magnetic Resonance Imaging (MRI) has been used to assess prostate motion, as it provides the required soft tissue contrast (24). With MR-linacs now commercially available, the soft tissue prostate intrafraction motion may be monitored in real time during treatment on these machines (9, 25–27).

Side effects from radiotherapy for prostate cancer could be further reduced with dynamic tracking and gating, as well as improving dose coverage and conformality (28, 29). Improving these aspects may allow a reduction in treatment margins, which would result in a potential reduction in dose to surrounding organs at risk (OAR). Introduction of techniques to allow a reduction of treatment margins would have benefit for all treatment fractionations, rather than only the stereotactic regimens for which these techniques are currently used to reduce the effect of intrafraction motion. This study implemented an in-house developed position monitoring software, SeedTracker, for conventional fractionation prostate radiotherapy, and studied the dosimetric impact of intrafraction motion.

## Methods

### Patient data

Patient data for this study was sourced from an ethics approved prospective clinical trial (ACTRN12618001421224). The study cohort was 30 prostate cancer patients with treatment to the prostate only, or both the prostate and whole pelvis depending on the disease stage. The key details of the patient cohort are shown in **Table 1**. The inclusion criteria for this study was prostate cancer patients receiving definitive radiotherapy treatment for prostate within Liverpool and Macarthur Cancer Therapy Centers, and patients implanted with three prostate radio opaque markers were eligible. The only study exclusion criteria were patients with hip prostheses. In the patient recruitment for the study, prostate cancer patients with implanted seeds were considered, and no specific restriction or preference was given to either prostate only or prostate with pelvic node patients. The equal split of prostate and prostate with pelvic nodes patients in this study (**Table 1**) is a coincidence and due to the result of our patient population at the time of study.

**Table 1:** Key patient characteristics of the study cohort.

Age (years)	Minimum	Maximum	Median
	60	84	77
	Prostate	Prostate and Pelvic Nodes	
Number of patients	15	15	
Dose Fractionation	60Gy/20 fractions	60Gy/20 fractions for prostate (One Patient 78Gy/39 fractions)	
		45Gy/20 fractions for Nodal volume (One Patient 54Gy/39 fractions)	
PTV margin	7mm	7mm	
Position monitoring tolerance and number of patients treated			
5mm	6	4	
4mm	7	3	
3mm	2	8	

### Plan and treatment delivery

Patients treated in this study followed departmental protocols for treatment planning and treatment delivery, utilizing CT based treatment planning and VMAT treatment delivery. Treatment plans were generated using Pinnacle treatment planning system using 6MV beam model for Elekta linac with Agility treatment head. For the prostate only cases, the treatment plans were generated using either single or dual full arcs depending on the plan requirement. Similarly, for prostate with pelvic nodes cases either two full arcs or 2 full arcs and one partial arc was used. The target and OARs dose objectives used for plan evaluation were given in **Table 2**. Pretreatment patient position was verified utilizing cone beam computed tomography (CBCT) prior to each treatment fraction with online correction to the implanted gold fiducial markers. The beam ON time and individual treatment fraction times of each patient were derived from the Mosaiq record and verify system.

**Table 2:** The clinical dose volume objectives of prostate and prostate with pelvic nodes plans.

Target Volume/OARs	Dose volume objectives (Dx/Vy)	
	Metric	Goal
Prostate		
PTV	D95	>60 Gy
	D50	60-62.6 Gy
	D2	<63 Gy
CTV	D99	>60Gy
Pelvis nodes		
PTV	D95	>42.75 Gy
CTV	D95	>45 Gy
OARs		
Rectum	V31	<50%
	V46	<30%
	V54	<10%
	V57.5	<5%
Sigmoid	V31	<50%
	V38	<40%
Bladder	V38	<40%
	V46	<30%
Femur	V31	<10%
Bowel Bag	V42.5	<10cc
	V40	<200cc
	V50	<5%

Dx = Dose received by x % of volume, Vy = Volume receiving y Gy of dose.

### Real-time position monitoring and tolerance criteria

An in-house developed position monitoring software, SeedTracker, was used for prostate online position monitoring. The SeedTracker system reads the planar x-ray images acquired during treatment and detects the position of fiducial markers implanted in the prostate and compares against the planned position. If the position of the markers deviates beyond the preset tolerance limits the system will alert the treating staff to interrupt the treatment delivery. The technical details on the SeedTracker system can be found elsewhere (22, 23, 30). To minimise the radiation dose from imaging, images were acquired at gantry angle intervals of 9°, possible with the intrafraction Cone Beam CT (IF-CBCT) acquisition in the Elekta XVI system. In the event of position deviations during treatment delivery, the treatment beam and image acquisition was interrupted manually by the treating staff and the 3D position offset was determined by a variable angle stereoscopic method available in SeedTracker using the last acquired IF-CBCT projection image and an additional planar image acquired at a 45° gantry angle separation (30). These functionalities were developed and incorporated within the SeedTracker system to enable the treatment interruption and resumption with IF-CBCT based image acquisition for online position monitoring.

The position tolerance for online verification was reduced progressively from 5mm, 4mm and finally 3mm to limit the unexpected increase in the treatment interruption that may disrupt routine workflow and the treatment machine schedule. The tolerance is considered for position deviations in any of the following directions: superior, inferior, left, right, anterior, and posterior. Ten patients were recruited and treated within each tolerance cohort. **Table 1** presents the distribution of patients treated in each tolerance cohort, specifying the number of patients in the ‘prostate only’ and ‘prostate and whole pelvis’ categories.

### Dosimetric assessment

The dose delivered to the target volumes and OARs was investigated using the SeedTracker system and the voxel-shift method (31, 32) In the “Corrected” scenario, the dose delivered with position corrections applied to observed position deviations was assessed. This was achieved by incorporating the residual position deviations below the action threshold into the three-dimensional (3D) dose distribution of the Volumetric Modulated Arc Therapy (VMAT) arc in each treatment fraction. On the other hand, in the “Not corrected” scenario, the dose that would have been delivered without monitoring was assessed through the following steps:

* In treatment fractions where position deviations did not occur, the residual position errors were incorporated into the VMAT arcs, similar to the corrected scenario.
* In cases where position deviations occurred at the start of the treatment, the observed position deviation was incorporated into the entire treatment fraction.
* In cases where position deviations occurred during the delivery of the treatment, the residual error calculated up to the fraction of treatment delivery was incorporated into the 3D dose distribution of the control points (CPs) of the VMAT arc up to the gantry angle of the position deviation event. For the remaining duration of the treatment fraction, the magnitude of the position deviation that triggered the event was incorporated into the CPs’ dose of the VMAT arc.

These steps allowed for the assessment of the dose that would have been delivered without monitoring the position deviations during treatment. The following target volumes and OAR dose volume histogram (DVH) metrics were used for the assessment:

* Prostate- CTV (CTVp) and node CTV (CTVn) –Dose received by 99% of volume (D99)
* Prostate- PTV (PTVp) and node PTV (PTVn) –Dose received by 98% of volume (D98)
* Rectum and Bladder – Volume receiving 60Gy (V60)

The actual delivered dose with position correction and the dose that would have been delivered without correction for the observed position deviations was compared with the original planned dose. The one-way analysis of variance (ANOVA), Tukey’s honestly significant difference (HSD) test was used to assess the significance of difference between the planned and delivered dose with and without position corrections.

## Results

### Treatment fraction time

**Table 3** displays the mean (standard deviation-SD) beam ON time and mean (SD) treatment fraction time for patients treated with prostate-only and prostate with pelvic nodes within the 5mm, 4mm, and 3mm tolerance cohorts. On average, the beam ON time for Prostate with pelvic nodes increased by 0.3mins compared to prostate only treatment. Reducing the position tolerance leads to an overall increase in treatment fraction time for both types of treatments. Specifically, when the tolerance is decreased from 5mm to 4mm, there is a maximum increase in mean treatment time of 0.5 minutes for prostate-only treatments. Similarly, in prostate with pelvic nodes treatments, the mean treatment time increases by 1 minute when the tolerance is reduced from 5mm to 3mm (**Table 3**).

**Table 3:** The mean beam ON and treatment fraction time of prostate only and prostate with pelvic nodes treatments.

Treatment site	Mean (SD) Beam ON time (minutes)	Mean (SD) treatment fraction time in individual tolerance cohorts (minutes)		
		5mm	4mm	3mm
Prostate	2.2 (0.2)	6.7 (2.7)	7.2 (3.2)	7.0 (2.4)
Prostate + pelvic nodes	2.5 (0.3)	9.7 (3.5)	10.0 (4.2)	10.7 (3.2)

### Position deviations and gating events

The mean (range) position deviations in the Left-Right (LR), Anterior-Posterior (AP), and Superior-Inferior (SI) directions for each tolerance criterion are presented in **Table 4A**. The overall mean (range) deviations were -0.4 (-7.2 – 5.3) mm in LR, -0.9 (-6.1 – 15.6) mm in AP, and -1.7 (-7.0 – 6.1) mm in SI directions. The percentage of position deviations in each direction that required table corrections within the respective tolerance cohorts is shown in **Table 4B**. Across all patients, 18.2% of position deviations in LR, 44.8% in AP, and 37.0% in SI directions triggered table corrections. The distribution of position correction directions for LR, AP, and SI is presented in **Table 4C**. There was a relatively higher percentage of position deviations in the left direction (56.8%) and posterior direction (56%) compared to the right direction (43.2%) and anterior direction (44%).

Table 4AThe mean (range) magnitude of position deviation that triggered a gating event.

**Table:** 

Tolerance cohort	Mean (range) mm		
	LR	AP	SI
5mm	5.3	-3.1 (-6.1 – 15.6)	-5.7 (-6.7 – -5.1)
4mm	-3.5 (-7.2 – 4.6)	3.5 (-5.7 – 5.8)	2.1 (-5.4 – 8.1)
3mm	-2.9 (-4.6 – 3.0)	-1.6 (-7.0 – 5.4)	2.2 (-3.5 – 6.1)
Overall	-0.4 (-7.2 – 5.3)	-0.9 (-6.1 – 15.6)	-1.7 (-7.0 – 6.1)

**Table 4B.** The percentage of position corrections observed in LR, AP and SI directions.

Tolerance Cohort	Direction of position correction		
	LR	AP	SI
5mm	17.5%	37.5%	45%
4mm	22.7%	40.0%	37.3%
3mm	14.8%	52.3%	32.9%
Overall	18.2%	44.8%	37.0%

**Table 4C.** The orientation of position correction in LR, AP and SI directions.

Directionality of position deviation					
Left	Right	Ant	Post	Sup	Inf
56.8%	43.2%	44.0%	56.0%	49.3%	50.7%

**Figure 1:**
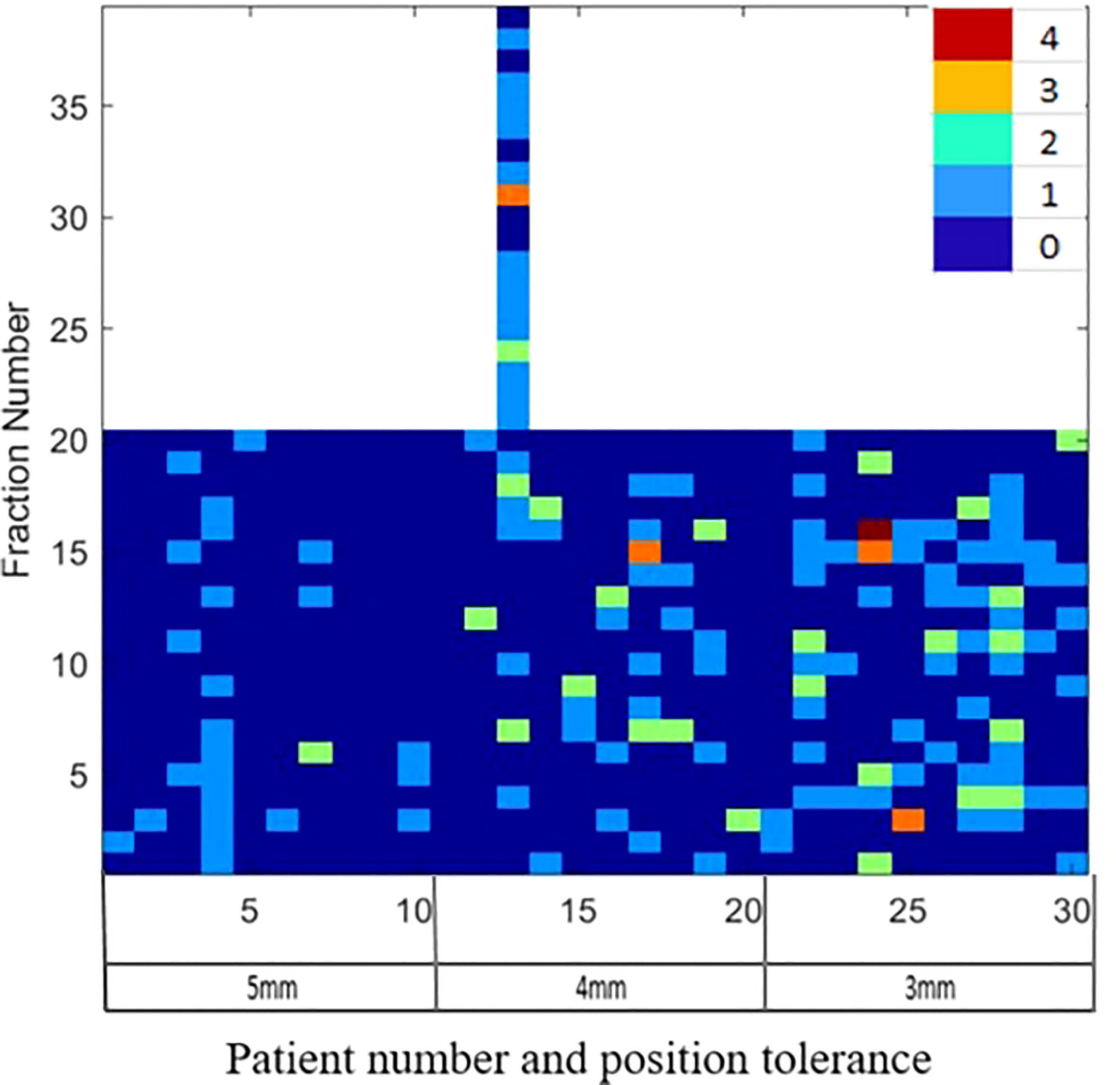
The gating events observed in the individual treatment fractions for patients treated with 5mm, 4mm and 3mm position tolerance criteria.

**Figure 1** illustrates the distribution of gating events observed in each of the treatment fractions. Among the 30 patients, 27 experienced at least one gating event that required a position correction. A total of 177 instances of position deviation were detected, occurring in 146 out of 619 treatment fractions. Among these occurrences, 111 treatment fractions had 1 gating event, 25 had 2 gating events, 2 had 3 gating events, and 1 had 4 gating events. The frequency of gating events is shown to increase as the position tolerance for monitoring decreases, as depicted in **Figure 1**.

Among all the position deviations observed, 40% were identified before the start of the first treatment arc, indicating that these deviations were detected prior to the initiation of treatment. In cases where the treatment plan consisted of multiple arcs, 39% of position deviations were detected before the start of the second treatment arc. The remaining 21% of position deviations were detected during the delivery of the treatment arc. When a 5mm tolerance was applied, a total of 26 position deviation events were identified in 200 treatment fractions (in 13% of fractions). With a reduced tolerance of 4mm, the number of events increased to 66, occurring in 219 treatment fractions (in 24% of fractions). Further decreasing the tolerance to 3mm resulted in 85 position deviation events observed in 200 treatment fractions (in 33% of fractions).

### Target position during treatment delivery

**Figure 2:**
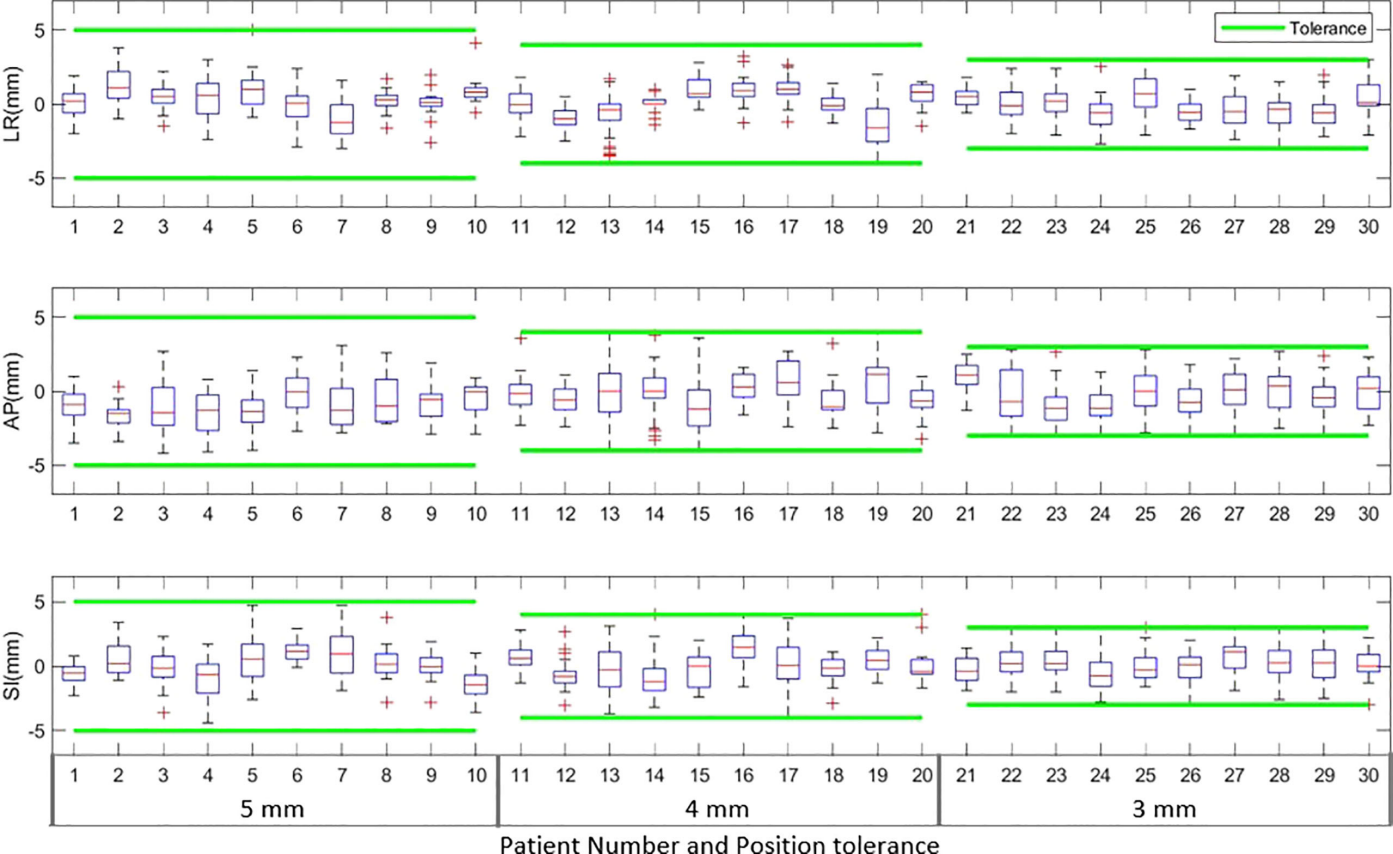
The target position in left-right (LR), anterior-posterior (AP) and superior-inferior (SI) directions during the actual treatment delivery for patients treated with 5mm, 4mm and 3mm tolerance criteria. The green lines in the plot show the tolerance band for each tolerance level.

The boxplots in **Figure 2** show the target position after correcting for applied to observed position deviations in LR, AP, and SI directions during treatment delivery for each patient. The green lines in the figure represent the tolerance criteria used in each of the patient treatments. The mean ± SD and range of target position in LR, AP, and SI directions during treatment delivery is shown in **Table 5A**. As illustrated in the **Figure 2** and **Table 5A**, with monitoring and position corrections applied the target was maintained well within the specified tolerance limits in each direction. The mean position of the target was maintained within ±1mm of the reference position in each direction (**Table 5A**).**Table 5B** shows the overall percentage of treatment fractions in which the prostate position difference was greater than 5mm, 4mm, and 3mm. In all the treatment fraction of the study cohort the prostate position deviated greater than 5mm, 4mm, and 3mm from the reference position in 13%, 23%, and 42% of fractions, respectively.

Table 5AThe mean target position during actual treatment delivery.

**Table:** 

Tolerance cohort	Mean±SD (range) mm		
	LR	AP	SI
5 mm	0.3±0.2 (-3.0 – 4.8)	-0.9±0.3 (-4.2 – 3.1)	0.0±0.3 (-4.4 – 4.7)
4 mm	0.1±0.3 (-3.9 – 3.2)	-0.1±0.4 (-4.0 – 3.9)	0.1±0.3 (-3.9 – 4.0)
3 mm	-0.1±0.2 (-3.0 – 3.0)	-0.2±0.2 (-3.0 – 2.8)	0.1±0.2 (-3.0 – 3.0)

**Table 5B.** The percentage of treatment fractions in which the prostate position deviation greater than 5mm, 4mm and 3mm in studied cohort of patients.

Prostate position deviation	% of fractions
≥5 mm	13 %
≥4 mm	23 %
≥3 mm	42 %

### Target dose

**Figure 3:**
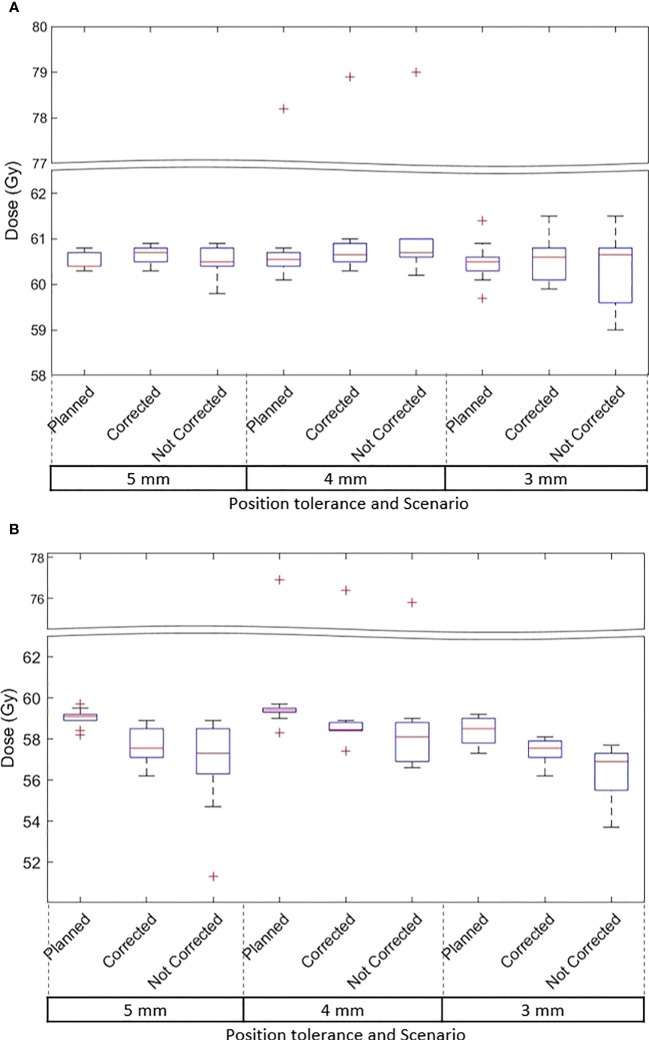
**(A)** The CTVp D99 and **(B)** PTVp D98 of the original plan, actual delivered treatment (with corrections performed for position deviations-Corrected) and delivery without corrections for patients treated with 5mm,4mm and 3mm position tolerance.

**Figure 4:**
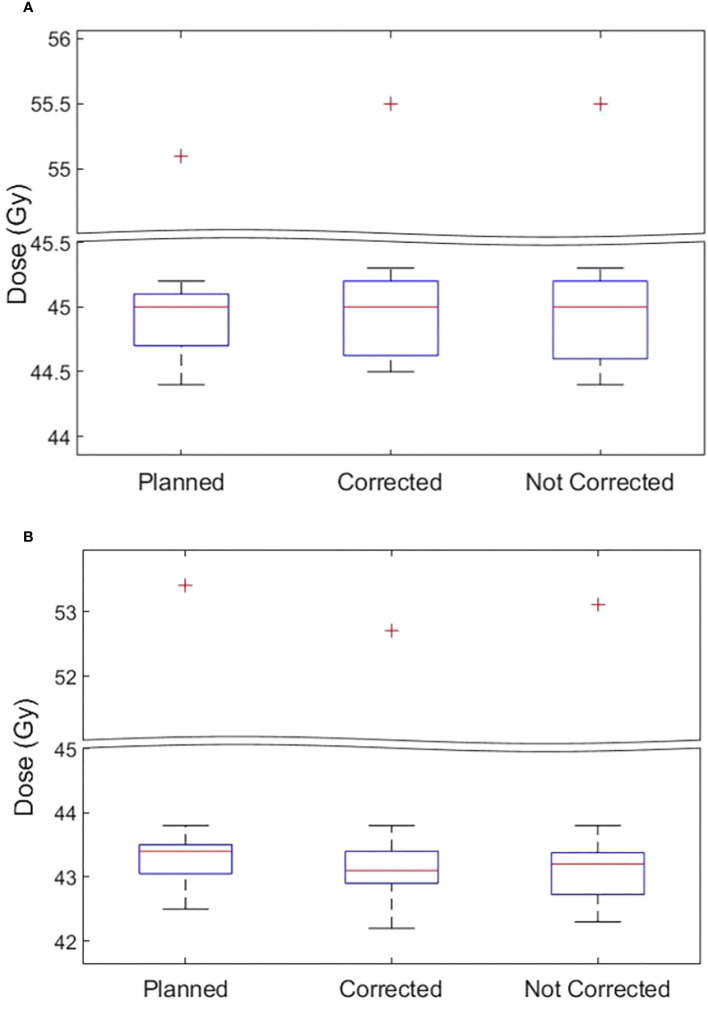
**(A)** The CTVn D99 and **(B)** PTVn D98 of the original plan, actual delivered treatment (with corrections performed for position deviations-Corrected) and delivery without corrections for patients treated in all tolerance cohorts.

The D99 to CTVp and D98 to PTVp of the original plan and treatment delivered with position corrections performed in patients treated with each tolerance cohort are shown in **Figures 3A, B** respectively. The dose that would have been delivered to the CTVp and PTVp without position corrections is also shown in the same figures. Similar metrics for CTVn and PTVn for patients treated in all tolerance cohorts are shown in **Figure 4**. The mean, standard deviation (SD), minimum and maximum difference between planned and delivered dose with and without position corrections in each tolerance cohorts for target volumes are shown in **Table 6**. The ANOVA, Tukey’s HSD test statistic is also shown in the same table. If the treatment was delivered without monitoring and position corrections the CTVp D99 would have been reduced by a maximum value of up to 1.3 Gy compared to the planned dose; with position corrections this difference was reduced to 0.3 Gy (**Table 6**). Similarly, if the treatment was delivered without position corrections the PTVp D98 would have been reduced by a maximum value of 7.6 Gy; with position corrections, this difference was reduced to 2.2 Gy. Overall, the mean(SD) D99 difference between the planned and treatment delivered without position corrections and treatment delivered with position corrections for CTVp was 0.1(0.2) Gy, and 0.0(0.4) Gy respectively. The corresponding difference for CTVn was 0.0(0.2) Gy and -0.1(0.2) Gy respectively. There was no significant difference between the planned and delivered dose to target volumes (**Table 6**) with and without corrections (p>0.05).

**Table 6:** The target volume dose difference between the original plan and the treatment delivered with and without corrections for position deviations.

Structure and DVH metric	Tolerance cohort	Dose difference (Gy)						ANOVA, Tukey’s HSD test results
		Delivery with corrections			Delivery without corrections			
		Mean (SD)	Min	Max	Mean (SD)	Min	Max	
CTVp D99	5mm	0.1 (0.2)	-0.1	0.4	0.0 (0.3)	-0.6	0.5	f-ratio=0.0093 p=0.99
	4mm	0.2 (0.2)	-0.2	0.7	0.2 (0.3)	-0.3	0.8	
	3mm	0.0 (0.2)	-0.3	0.2	-0.2 (0.5)	-1.3	0.2	
PTVp D98	5mm	-1.3 (0.7)	-2.2	-0.6	-2.2 (2.2)	-7.6	-0.6	f-ratio=2.114 p=0.13
	4mm	-0.9 (0.4)	-2.0	-0.5	-1.4 (0.8)	-2.8	0.8	
	3mm	-0.9 (0.4)	-1.7	-0.2	-2.0 (1.0)	-3.6	-0.4	
CTVn D99	5mm	0.1 (0.1)	0.0	0.2	0.1 (0.1)	-0.1	0.2	f-ratio=0.0019 p = 0.99
	4mm	0.1 (0.4)	-0.3	0.4	0.1 (0.4)	-0.3	0.4	
	3mm	0.1 (0.1)	0.0	0.2	0.0 (0.1)	-0.1	0.1	
PTVn D98	5mm	-0.3 (0.3)	-0.7	0.0	-0.1 (0.1)	-0.2	0.0	f-ratio=0.0405 p = 0.96
	4mm	-0.5 (0.2)	-0.7	0.3	-0.3 (0.1)	-0.3	-0.2	
	3mm	-0.3 (0.3)	-0.7	-0.1	-0.1 (0.1)	-0.3	0.0	

### OAR dose

**Figure 5:**
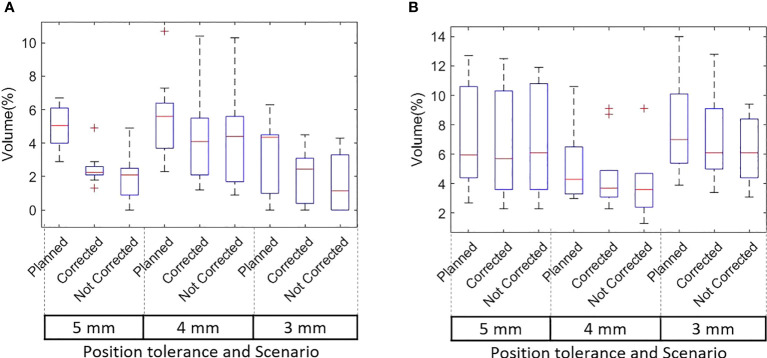
**(A)** Rectum and **(B)** Bladder V60 of the original plan, actual delivered treatment (with corrections performed for position deviations) and delivery without corrections for patients treated with 5mm, 4mm and 3mm position tolerance.

**Figures 5A, B** shows the rectum and bladder V60 of the original plan and actual treatment delivered with the correction of position deviations. The dose that would have been delivered without position corrections is also shown in the same figures. The difference in V60 to the bladder and rectum between the planned and delivered dose with and without position corrections, and ANOVA,Tukey’s HSD test statistic is shown in **Table 7**. Without correcting for position deviations the V60 to the rectum increased by a maximum of 1.0% compared to the planned dose. With position correction, this was reduced to 0.5%. There was no ststistically significant difference between planned and delivered V60 to bladder (p>0.05). A one way ANOVA revealed that there was a statistically significant difference of V60 to the rectum between the planned and delivered dose with and without position corrections (**Table 7**). Tukey’s HSD Test for multiple comparisons found that the mean value of rectum V60 was significantly different between planned and both delivery with and without position corrections (p<0.05). There was no significant difference in rectum V60 between the delivery with and without corrections (p>0.05).

**Table 7:** The rectum and bladder dose-volume difference between the original plan and the treatment delivered with and without corrections for position deviations.

Structure and DVH metric	Tolerance cohort	Volume difference (% Volume)						ANOVA, Tukey’s HSD test results
		Delivery with corrections			Delivery without corrections			
		Mean (SD)	Min	Max	Mean (SD)	Min	Max	
Rectum V60	5mm	-2.5 (1.1)	-3.8	-0.3	-3.0 (1.8)	-6.5	-0.1	f-ratio =6.7539 p = 0.001 Planned : Corrected p =0.012 Planned : Not corrected p=0.003 Corrected : Not corrected p =0.882
	4mm	-1.2 (1.1)	-3.4	0.5	-1.1 (1.5)	-3.9	1.0	
	3mm	-1.4 (1.8)	-5.8	0.3	-1.8 (1.9)	-5.8	0.1	
Bladder V60	5mm	-0.3 (0.6)	-1.3	2.1	-0.1 (1.0)	-1.3	2.1	f-ratio =0.5881 p = 0.557
	4mm	-0.7 (0.8)	-1.9	0.3	-1.0 (1.1)	-3.0	0.7	
	3mm	-0.8 (0.5)	-1.7	-0.2	-1.4 (1.4)	-4.9	-0.2	

## Discussion

Prostate intrafraction motion introduces uncertainty and requires the addition of an intrafraction motion margin to ensure adequate dose coverage to the tumor. Several methods of intrafraction prostate position monitoring have been investigated in the literature, including electromagnetic tracking using implanted radiofrequency (RF) beacons, implanted radiopaque marker-based tracking using cine portal images acquired using Electronic Portal Imaging devices (EPIDs), pre and post treatment CBCT images, continuous fluoroscopic imaging and the use of online ultrasound images (6, 33–36). Whilst real-time prostate position monitoring is widely implemented in prostate stereotactic body radiotherapy (SBRT), the implementation in conventional fractionation treatment is limited owing to the requirement of additional monitoring systems, increased work resulting from the gating and position correction events and resulting additional cost involved. In this work, real-time position monitoring in conventional fractionation prostate treatment was achieved using an imaging system used for pretreatment position verification available on the Elekta linear accelerator. The real-time position monitoring and position correction workflows were developed to fit within the intrafraction CBCT image acquisition workflow for treatment with an Elekta linear accelerator (37). The implemented real-time monitoring enabled the detection of position deviations outside the specified position tolerance and allowed for position corrections and improved treatment delivery accuracy.

Langen et al. quantified the intrafraction motion of the prostate using Calypso electromagnetic tracking system and observed the prostate 3D displacement of > 10mm in 15.2% of the tracking sessions of one of the patients they analysed (38). In their analysis, they have not observed a lateral movement >5mm. In contrast, this study observed a maximum LR offset of 7.2mm (**Table 4A**). Shimizu et al. analyzed the intrafraction prostate position data of 20 patients treated using a real-time tumor tracking radiotherapy system (39). Based on their data, LR and AP displacements of >10mm and SI displacement of >15mm were observed in some of the fractions. The range of the position deviations observed in our study agrees with the magnitude of displacement reported by Shimizu et al. (**Table 4A**).

Based on 550 treatment sessions of prostate tracking data, Shimizu et al. reported that at 10 minutes from the initial setup of each treatment, the incidence of table correction required was 14.2%, 12.3%, and 5.0% in AP, SI, and LR directions (39). Our data qualitatively agrees with this (**Table 4B**). Based on the position deviation events observed in our data the majority of position deviations (44.8%) were observed in the AP direction followed by the SI direction (37.0%). The corrections along the LR direction showed the least (18.2%) of all three directions. Whilst the relative distribution of corrections in the AP and SI directions show a similar trend in the 4mm and 5mm tolerance cohorts, in the 3mm tolerance cohort the corrections in the SI direction were relatively higher compared to the AP direction (**Table 4B**). Regarding the direction of the displacement along SI and AP directions, Langen et al. reported that the prostate is approximately twice more likely to move inferiorly than superiorly, and posteriorly more so than anteriorly in the events where displacements are >3mm. Our data showed a similar trend qualitatively in SI and AP directions (**Table 4C**), however, the magnitude of the difference is relatively less compared to the results reported by Langen et al.

Kupelian et al. used the Calypso electromagnetic tracking system to monitor the prostate position in 41 patients and reported a prostate displacement of ≥ 3mm and ≥ 5mm for a cumulative duration of at least 30s was observed in 41% and 15% of the treatment sessions (35). In our study, a prostate displacement of ≥ 3mm and ≥ 5mm was observed in 42% and 13% of treatment fractions which is in close agreement with the results reported by Kupelian et al. (**Table 5B**). The number of position corrections required to keep the prostate within the specified position tolerance increased as tighter tolerances were used for treatment. With the 3mm position tolerance criteria approximately 1 in every 2 fractions required a table correction to remain within tolerance. With the 4mm and 5mm position tolerance criteria, the required table corrections were reduced to 1 in 4 and 1 in 8 fractions respectively (**Table 5B**). The step-wise reduction of tolerance criteria implemented in this study enabled the treating staff to gradually adjust the routine treatment workflow with the integration of the real-time position verification without introducing a significant burden on the workforce.

Langen et al. reported that at an individual patient level, a prostate displacement >3mm was observed in a maximum of 75% of the treatment sessions (38). In our data, at an individual patient level, a maximum of 75% of treatment fractions required position corrections (**Figure 1** patient 22). At the 4mm and 5mm tolerance criteria, this was reduced to 55% (**Figure 2**, patients 13 and 4). About 40% of the position deviations detected in this study occurred at the start of the treatment after initial CBCT-based verification which has the highest impact on the accuracy of the delivered dose. The process of CBCT image reconstruction, image registration and verification, and treatment parameter checks before the start of treatment requires considerable time between the CBCT image acquisition and treatment start. The factors such as bladder filling, peristalsis, and pelvic movement could attribute to this observed position deviation just before the start of treatment. Langen et al. reported that the probability of prostate displacement >3mm increases by about 12.5% and 25% after initial alignment of 5 minutes and 10 minutes respectively (38). In actual treatment delivery with position corrections, a mean ± SD target position accuracy of -0.9 ± 0.3mm was achieved in this study (**Table 5A**).

An isotropic PTV margin of 7mm was used for all the patients treated in this study as per departmental protocol. Overall, the treatment delivered with position corrections showed an improved agreement of CTVp D99 and PTVp D98 with the planned dose in comparison to the simulated treatment delivery without position corrections (**Figures 3A**, **Table 6**). The treatment without position corrections applied showed a maximum CTVp D99 underdosage of 1.3Gy (-2.2% of the planned dose) however with real-time image guidance this difference was reduced to 0.3Gy (-0.5%). The variations in CTVp and PTVp doses are relatively high in patients treated in the 3mm tolerance cohort (**Figures 3A**). Eight of the 10 patients treated in the 3mm cohort received treatment for both prostate and nodes (**Table 1**). The gradients in planned dose due to the multilevel dose prescription could contribute to this variation. The prostate only and prostate and whole pelvis patient distribution within each tolerance cohort was not controlled in this study as the recruitment occurred as per the inflow of prostate cancer patients to the clinic at the time of this study. There was no statistically significant difference between planned and delivered dose to target volumes (CTVp, CTVn, PTVp and PTVn) with and without position corrections. Whilst the 7mm PTV margin used in this study provided adequate coverage in the majority of the patients, in some patients this margin was not enough and impacted the dose to the CTVp adversely. Future real-time multi-target MLC tracking approaches could reduce the suboptimal dose delivery to static nodal volume while correcting for intrafraction prostate motion (40). Keall et al. assessed intrafraction prostate motion and its impact on the CTV dose in prostate SBRT using a kilovoltage intrafraction imaging system (41). Their results showed that the treatment without correcting for intrafraction position deviation would result in prostate D98 doses 5% less than planned in 5.5% of the treatment fractions. Faccenda et al. conducted a study on the dosimetric impact of intrafraction motion using the Raypilot system in prostate SBRT. They reported that when prostate position deviations were corrected, the mean (range) relative dose differences between delivered and planned treatments for CTV D99% were -3.0% [-18.5-2.8] (42).The delivered CTV dose analysis of our study qualitatively agrees with the results reported by Keall et al. (41). However, the quantitative differences are expected due to a smaller PTV margin and the lower number of treatment fractions in SBRT.

The estimation of the actual delivered dose provides an accurate surrogate to estimate the treatment outcomes (43). The delivered dose estimation performed in this study incorporates the actual prostate position during treatment delivery, which provides a better evaluation of the delivered dose to target and OARs rather than assuming the planned dose as the delivered dose. The rectum and bladder V60 of the treatment delivered with position corrections showed consistently better agreement with the planned dose in comparison to the simulated delivery without position corrections (**Figure 5**, **Table 7**). The delivery without position correction would have resulted in the rectum V60 increasing by a maximum of up to 1.0% from the planned volume. With position correction, this difference was reduced to 0.5%. The planned V60 to the rectum was statistically different when compared to both delivery with and without corrections. However, there was no statistically significant difference between corrected and not corrected treatment scenarios (**Table 7**). The overlap of PTV with the rectum, the high dose gradient in the planned dose, and residual position error after correction could be the reasons for differences between the planned and delivered doses. The 7mm PTV margin used in our clinic was sufficient to compensate for the observed intrafraction motion, and there was no statistically significant difference in the delivered target volume and OARs dose between the corrected and not corrected scenarios. Studies have demonstrated the potential reduction in toxicity with a reduction in the margin during prostate radiotherapy, particularly in specialized radiotherapy delivery systems such as the RTRT system and MRI linac (44, 45). The successful implementation of real-time position monitoring in a general-purpose linac for conventional fractionation prostate radiotherapy will enable the reduction of the CTV-PTV margin, thereby reducing treatment-related toxicity.

The delivered dose estimation performed in this study is based on the planning CT dataset which does not account for day-to-day variation in the bladder and rectum size and shape (46, 47). Studies have shown that there can be deformation of the prostate during the radiotherapy course (48, 49). Based on CT images acquired at multiple time points, Lebesque et al. quantified the rectum and bladder wall variations of up to 9% and 17% during the course of radiotherapy (47). Whilst the target volume and OARs defined on the daily pretreatment verification CBCT images would have improved the delivered dose estimation accuracy in our study, the accurate contouring of these structures on verification CBCT images is challenging and introduces more uncertainties. Lutgendorf -Cauig et al. reported larger inter observer variation of prostate contoured on CBCT (conformity index (CI): 0.57 ± 0.09) compared to MRI (CI: 0.66 ± 0.12) and CT (CI: 0.72 ± 0.07) (50).

Real-time position monitoring and correction for intrafraction position deviations are paramount in prostate SBRT due to tighter margins, increased dose per fraction, and relatively longer treatment time. In conventional fractionation prostate radiotherapy, the use of a wide CTV-PTV margin generally accounts for geometric uncertainties arising from intrafraction position deviations. The ability to correct for intrafraction position deviations allows for the possibility of reducing the magnitude of the CTV-PTV margin used in conventional fractionation prostate radiotherapy, thereby reducing the dose to OARs and potentially resulting in reduced treatment-related toxicity. In this study, we gradually reduced the tolerance for real-time monitoring from 5mm to 3mm to avoid major disturbances to patient scheduling that may arise from an increased frequency of position correction events with tighter tolerance. The 7mm CTV-PTV margin used in our clinics shows that tighter tolerance for position monitoring does not impact the accuracy of CTV dose delivery. However, the selection of a suitable position tolerance depends on the magnitude of the CTV-PTV margin used in the clinic. Our study demonstrates the feasibility of using tighter position tolerance for real-time monitoring in conventional prostate RT, which would allow for reduced CTV-PTV margins.

The important contribution of this study is the successful implementation of real-time image guidance for prostate radiotherapy using an in-house developed position monitoring software which utilizes the x-ray imaging system commonly available on standard linacs. To our knowledge, this is the first prospective study that reported the use of a standard imaging system on a conventional linac for online position correction in conventional fractionation prostate radiotherapy. The workflow developed seamlessly integrated with the IF-CBCT imaging option available in Elekta linacs and enabled real-time monitoring without additional demand on logistics and workforce. The additional radiation dose resulting from online imaging was reduced as low as possible through reduced imaging frequency and a smaller field aperture. The improved treatment delivery accuracy with online position monitoring demonstrated in this study would enable the safe reduction of the PTV margin which would potentially reduce the dose to OARs and may improve the patient quality of life.

## Conclusions

Online target position monitoring for conventional fractionation prostate radiotherapy was successfully implemented on a standard linac using an in-house developed position monitoring software. Treatment with online position corrections resulted in an improvement in the accuracy of dose delivered to prostate target volume.

## Data availability statement

The original contributions presented in the study are included in the article/supplementary material. Further inquiries can be directed to the corresponding author.

## Ethics statement

The studies involving human participants were reviewed and approved by Human Research Ethics Committee, South Western Sydney Local Health District, Liverpool, Sydney, Australia. The patients/participants provided their written informed consent to participate in this study.

## Author contributions

SA and MS developed the study concept SA drafted the manuscript. VD and KW contributed to the protocol development and study design. TY, PC and CT and MU contributed to the study administration and data collection. All authors contributed to the article and approved the submitted version.
